# Shenyuan granules improve cellular senescence through Klotho-mediated p16/p21 signaling pathway in diabetic kidney disease

**DOI:** 10.3389/fmed.2025.1627412

**Published:** 2025-07-31

**Authors:** Xinyuan Guo, Siyang Zhang, Qingqing Sun, Huimeng Li, Lan Wang

**Affiliations:** ^1^School of Traditional Chinese Medicine, Hubei University of Chinese Medicine, Wuhan, China; ^2^The First Clinical College, Hubei University of Chinese Medicine, Wuhan, China; ^3^Hubei Provincial Hospital of Traditional Chinese Medicine, Affiliated Hospital of Hubei University of Chinese Medicine, Wuhan, China; ^4^Hubei Shizhen Laboratory, Wuhan, China; ^5^Hubei Key Laboratory of Theory and Application Research of Liver and Kidney in Traditional Chinese Medicine, Hubei Provincial Hospital of Traditional Chinese Medicine, Wuhan, China

**Keywords:** traditional Chinese medicine, Shenyuan granules, cellular senescence, Klotho, p16, p21

## Abstract

**Context:**

Shenyuan Granules (SYG), a traditional Chinese medicine preparation, are clinically used for treating chronic kidney diseases. However, the role of Klotho in modulating cellular senescence via the p16/p21 pathway and its involvement in the therapeutic effects of SYG in diabetic kidney disease (DKD) remains unclear.

**Objective:**

This study investigated the regulatory effects of SYG on the Klotho gene and their mechanisms in alleviating cellular senescence in DKD.

**Materials and methods:**

Utilizing an adenine-induced DKD model in *db/db* mice and AGE-stimulated HK-2 cells, this research assessed renal tissue for cellular senescence and pathological changes. Techniques such as SA-*β*-Gal, HE, and PAS staining were employed to observe these changes. The study also measured the expression levels of senescence-associated and anti-aging markers including Klotho, cyclin-dependent kinase inhibitor 2A (p16), cyclin-dependent kinase inhibitor p21 (p21), plasminogen activator inhibitor-1. Quantification of senescent cells was performed using SA-*β*-Gal staining, while mRNA and protein expressions were analyzed using immunofluorescence, real-time PCR, and Western blotting.

**Results:**

SYG treatment significantly improved renal function in db/db mice and alleviated histopathological lesions. SA-*β*-Gal staining demonstrated a marked decrease in senescent cell burden, while immunohistochemistry and Western blotting revealed downregulation of p16, p21, and PAI-1 and upregulation of Klotho expression (*p < 0.05*). *In vitro*, Klotho overexpression in AGE-stimulated HK-2 cells significantly suppressed senescence-associated markers and restored Lamin B1 expression. Similarly, treatment with SYG-containing serum effectively downregulated p16, p21, and PAI-1 while upregulating Klotho expression. These findings suggest that SYG attenuate renal cellular senescence by modulating the Klotho-mediated p16/p21 signaling pathway.

**Discussion:**

This study highlights the potential of SYG to alleviate cellular senescence in DKD by targeting the Klotho-mediated p16/p21 pathway. These findings provide a foundation for developing senescence-focused therapies in chronic kidney disease management.

## Graphical abstract

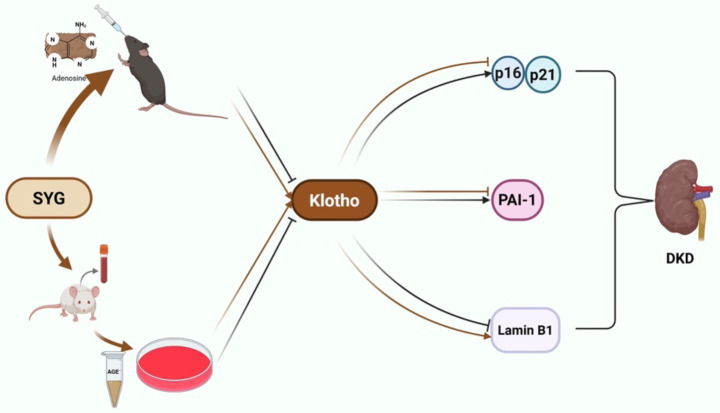

## 1 Introduction

Diabetes mellitus (DM) is a chronic disease caused by metabolic and endocrine disorders, with high global prevalence and mortality (1, 2). Diabetic kidney disease (DKD), one of the most common chronic microvascular complications of DM, is also a leading cause of end-stage renal disease (ESRD) (3). The severity of DKD is strongly influenced by the duration of diabetes, glycemic control, and genetic predisposition (4). Clinically, DKD is characterized by renal dysfunction or elevated urinary albumin excretion, with some patients exhibiting both features. With the rising prevalence of diabetes, the incidence of DKD has also significantly increased. Nevertheless, effective treatment options for DKD remain limited, representing a major threat to public health and patient quality of life (5, 6).

Cellular senescence is defined as an irreversible cell cycle arrest induced by various stresses such as oxidative stress, DNA damage, or telomere shortening (7). While initially considered a protective mechanism against malignant transformation, abnormal or excessive senescence disrupts tissue homeostasis and promotes the development of chronic diseases (8). Recent studies have demonstrated that cellular senescence plays a critical role in the onset and progression of various kidney diseases, including DKD (9–11). Senescent cells are often accompanied by a senescence-associated secretory phenotype (SASP), characterized by the overexpression of proinflammatory cytokines, chemokines, and matrix metalloproteinases. These factors aggravate tubular injury and interstitial fibrosis, thereby amplifying renal damage (12). Therefore, targeting cellular senescence and its related pathways represents a promising strategy for the treatment of DKD.

The Klotho gene, a well-recognized anti-aging factor, is predominantly expressed in the distal renal tubules. Klotho functions through both its membrane-bound and secreted forms, playing essential roles in calcium-phosphorus homeostasis, oxidative stress resistance, and antifibrotic activity (13). Studies have shown that Klotho expression is significantly reduced in DKD patients and animal models, which correlates with renal dysfunction and structural damage (14, 15). Although Klotho has been identified as a potential biomarker and therapeutic target for kidney diseases, its precise involvement in cellular senescence and related signaling pathways remains to be fully elucidated (15).

A growing body of evidence suggests that traditional Chinese medicine (TCM) is widely used in the treatment of renal diseases, including DKD (16–19). Shenyuan granules (SYG), a TCM formulation clinically used for the treatment of chronic kidney diseases, are composed of *Astragalus membranaceus* (Fisch. ex Link, Fabaceae), *Epimedium brevicornu* (Maxim., Berberidaceae), and processed *Rheum officinale* (Baillon, Polygonaceae).

Previous studies have demonstrated that SYG can improve renal function and attenuate pathological injury (20, 21). Astragalus, rich in flavonoids and saponins, exerts antioxidative, anti-inflammatory, and immunomodulatory effects that alleviate hyperglycemia-induced renal dysfunction (22, 23). Icariin, the major active component of Epimedium, has been shown to delay endothelial cell senescence via the PI3K/Akt-eNOS signaling pathway (24). Moreover, anthraquinones in Rheum, such as emodin and chrysophanol, not only possess antifibrotic effects but also inhibit cellular senescence by modulating the p53 signaling pathway (25, 26). However, it remains unclear whether Klotho is involved in SYG-mediated regulation of cellular senescence via the p16/p21 axis in DKD.

In this study, a DKD mouse model was induced using an adenine-containing diet, followed by intragastric administration of SYG. We aim to evaluate whether SYG can upregulate Klotho expression, mitigate renal damage, and delay cellular senescence. Furthermore, AGE-induced HK-2 cells were employed to analyze changes in Klotho expression and key senescence-associated molecules, providing further mechanistic insight into the anti-senescence effects of SYG in DKD.

## 2 Materials and methods

### 2.1 Chemicals and reagents

SYG (20230501, an in-hospital preparation from Hubei Provincial Hospital of Traditional Chinese Medicine, China) were used in this study. Klotho-overexpressing lentivirus was packaged and constructed by GeneChem Co., Ltd. (Suzhou, China). Primary antibodies included Klotho (rabbit, 1:1000, ab181373, RRID:AB_3694098) and p16 (rabbit, 1:1000, ab51243, RRID:AB_2059963) purchased from Abcam (Cambridge, MA, United States). Additional antibodies were purchased from Proteintech (Wuhan, China), including Klotho (mouse, 1:2000, 67331-1-Ig, RRID: AB_2882590), PAI-1 (mouse, 1:5000, 66261-1-Ig, RRID: AB_2881648), p16 (rabbit, 1:1000, 10883-1-AP, RRID: AB_2078303; rabbit, 1:1000, 28416-1-AP, RRID: AB_3086048), p21 (rabbit, 1:1000, 10355-1-AP, RRID: AB_2077682; rabbit, 1:1000, 28248-1-AP, RRID: AB_2881097), Lamin B1 (mouse, 1:5000, 66095-1-Ig, RRID: AB_11232208), *β*-actin (mouse, 1:20000, 66009-1-Ig, RRID: AB_2687938), and KIM-1(rabbit,1:10030948-1-AP, RRID: AB_3669790). The rabbit anti-β-actin antibody (1:10000, AF7018, RRID: AB_2839420) was purchased from Affinity Biosciences (Melbourne, Australia). Additionally, another Klotho antibody (rabbit, 1:100, CY7174, RRID: AB_3698752) was purchased from Abways (Shanghai, China) and used for immunohistochemical staining with improved specificity and clarity. For secondary antibodies, HRP-conjugated Affinipure Goat Anti-Mouse IgG (H + L) (1:5000, SA00001-1, RRID: AB_2722565) and HRP-conjugated Affinipure Goat Anti-Rabbit IgG (H + L) (1:5000, SA00001-2, RRID: AB_2722564) were obtained from Proteintech.

### 2.2 SYG in the treatment of adenine-induced DKD mice

Male C57BLKs/J db/db mice (34.51 ± 3.08 g, 7 weeks old) were purchased from GemPharmatech Biotechnology Co., Ltd. (Nanjing, Jiangsu, China, approval no. SCXK [Chuan] 2020-0034). The DKD model in *db/db* mice was established as described in previous publication (21). The *db/db* mice were randomly divided into three groups: DKD (model group), DKD + SYG-M (middle-dose SYG group, 3.0 g/kg/d), and DKD + SYG-H (high-dose SYG group, 6.0 g/kg/d) (*n* = 8/group). Additionally, CTL (*db/m* mice, *n* = 8). The DKD and SYG groups were fed a 0.2% adenine-containing diet, while the CTL was fed a standard diet. Mice in SYG-M were administered a suspension of SYG at a dose of 3.0 g/kg, and those in SYG-H received a suspension of 6.0 g/kg. Both suspensions were delivered via intragastric gavage at a volume of 20 mL/kg once daily. Mice in DKD and CTL were gavaged with an equal volume of double-distilled water (20 mL/kg) once daily. The treatments were continued for 12 consecutive weeks. After 12 weeks of administration, 24-h urine samples were collected from the mice using metabolic cages, during which the mice had free access to food and water. At the end of the study, mice were deeply anesthetized with 1.25% tribromoethanol (0.2 mL/10 g body weight) prior to collect blood and kidney tissues for subsequent experiments. Throughout the study, animal health was monitored daily, and humane endpoints were applied to minimize suffering, including euthanasia for mice with severe symptoms such as weight loss exceeding 20%, impaired mobility, or feeding difficulties. At the study endpoint, euthanasia was performed by cervical dislocation under deep anesthesia. All animal care and experimental procedures were approved by the Animal Ethics Committee of Hubei University of Chinese Medicine (approval no. HUCMS00304837).

### 2.3 Cell culture

HK-2 cells were obtained from the China Center for Type Culture Collection (CCTCC, GDC1502, China). The cells were cultured in DMEM-F12 medium containing 10% fetal bovine serum (FBS) (Gibco, Carlsbad, CA, United States) in a humidified incubator with 5% CO₂ at 37°C.

### 2.4 SYG serum preparation

Twelve male Sprague–Dawley (SD) rats (aged 6 weeks) were purchased from Hunan Silaike Jingda Laboratory Animal Co., Ltd. (Changsha, Hunan, China, approval no. SCXK [Xiang] 2019-0004), and were then weighed and randomly assigned to three groups based on body weight: blank serum group, low-dose SYG group, and high-dose SYG group (*n* = 4/group). The dosages for the rats were calculated based on clinical doses for humans. For the low-dose group, 10 g of SYG were dissolved in 29.4 mL of distilled water, while for the high-dose group, 10 g of SYG were dissolved in 14.7 mL of distilled water. The blank serum group received an equivalent volume of distilled water. All groups were administered 10 mL∙kg^−1^ of the respective solutions via intragastric gavage once daily for 7 consecutive days. On the 7th day, 1 h after the final gavage, the rats were anesthetized via intraperitoneal injection of 2.5% tribromoethanol at a dosage of 300 mg/kg body weight. Health monitoring was performed daily during the experiment, including assessments of body weight, food and water intake, activity, and physical appearance. If rats exhibited a weight loss greater than 20%, persistent anorexia, severe inactivity, or other signs of distress, humane endpoints would be applied. Blood samples (approximately 10 mL) were collected from the abdominal aorta under deep anesthesia. Euthanasia was achieved by exsanguination under anesthesia, following ethical standards to minimize animal suffering. The collected blood samples were centrifuged to obtain serum, aliquoted, and stored at −80°C for future use. All animal care and experimental procedures were approved by the Animal Ethics Committee of Wuhan HuaLianKe Biotechnology Co., Ltd. (approval number: HLK-20230925-001).

### 2.5 Cell viability assay

The cell viability of HK-2 cells was assessed using the CCK-8 assay kit (BS350A, Biosharp, Labgic Technology Co., Ltd., Beijing, China). HK-2 cells were seeded into 96-well white plates with a transparent bottom at a density of 10^4^ cells per well. The cells were divided into a control group and drug-containing serum groups with different serum concentrations (5, 10, 15, 20, 25, and 30%). After adherence, the cells were treated with serum-free medium for 24 h to induce starvation. Subsequently, the medium was discarded, and the cells were treated according to the experimental design and incubated for 72 h. After incubation, 10 μL of CCK-8 reagent was added to each well and incubated for approximately 2 h. The absorbance (A) of each well was measured at 450 nm using a microplate reader (VersaMax, Molecular Devices, United States).

### 2.6 Klotho overexpression and silencing

HK-2 cells in the logarithmic growth phase were seeded into 6-well plates at a density of 1 × 10^5^ cells per well. After attachment, cells were infected with lentivirus at a multiplicity of infection (MOI) of 5, with 1:200 diluted Polybrene added to each well. The medium was replaced 24 h post-infection. When cells reached approximately 80% confluency, they were trypsinized and transferred to 10 cm culture dishes. After attachment, 2 μg/mL puromycin (Beyotime, ST551, Shanghai, China) was added for selection, and cells were cultured for 3 weeks with medium changes every 2 days to eliminate uninfected cells.

When cell density reached approximately 70%, siRNA transfection was initiated. siRNA was diluted in serum-free medium, with 1.25 μL siRNA added to 125 μL medium (final concentration: 25 pmol), mixed thoroughly. Separately, 7.5 μL RNA transfection reagent was added to another 125 μL of medium and mixed. The siRNA and transfection reagent mixtures were combined at a 1:1 ratio, allowed to incubate for 5 min, and then 250 μL of the mixture was added to each well. The cells were cultured for an additional 72 h before sample collection.

### 2.7 Cell treatment with advanced glycation end product (AGE) and SYG

Patients with DM exhibit elevated levels of AGE, which can directly damage renal cells and tissues (27). In this study, HK-2 cells were stimulated with 100ug/mL AGE in the presence or absence of drug-containing serum from SYG. For mechanistic investigation, cells were divided into six groups: CTL, AGE, AGE + oe-NC, AGE + oe-Klotho, AGE + si-NC, and AGE + si-Klotho. For treatment evaluation, cells were grouped as follows: CTL, AGE + Blank Serum, AGE + SYG Serum, AGE + Blank Serum + oe-NC, and AGE + Blank Serum + oe-Klotho. The treated HK-2 cells were collected for subsequent experimental analysis.

### 2.8 Senescence-associated *β*-galactosidase (SA-β-Gal) staining

#### 2.8.1 Cell staining

The culture medium in the 24-well plate was discarded, and the cells were rinsed with PBS for 5 min. β-galactosidase staining fixative was added and incubated at room temperature for 15 min, followed by three washes with PBS, each for 5 min. After removing PBS, the staining working solution was prepared and added to the wells, ensuring the cells were fully covered. The cells were incubated in a 37°C incubator (without CO₂) overnight in the dark. After incubation, the staining solution was discarded, and the cells were gently rinsed twice with PBS. The staining results were observed and photographed for analysis.

#### 2.8.2 Tissue staining

Frozen kidney sections were equilibrated to room temperature, washed, fixed for 20 min, and immersed in staining working solution. The sections were incubated at 37°C overnight in the dark and mounted for observation and imaging.

### 2.9 Biochemical index detection

Serum samples from each group of mice were collected, and serum creatinine (Creatinine (Cr) Assay kit (sarcosine oxidase), C011-2-1) and blood urea nitrogen (Urea Assay Kit, C013-2-1) levels were measured according to the kit instructions. The kits were purchased from Nanjing Jiancheng Bioengineering Institute.

### 2.10 Light microscopy study

#### 2.10.1 Hematoxylin and eosin (HE) staining

Kidney tissues were fixed in 4% paraformaldehyde overnight, followed by dehydration through a graded ethanol series, clearing in xylene, embedding in paraffin, and sectioning. Sections were dewaxed, rehydrated through a descending ethanol series, and stained with hematoxylin. After rinsing with running tap water, sections were differentiated in 1% hydrochloric acid ethanol, rinsed again, and returned to blue. Subsequently, sections were counterstained with 1% eosin, dehydrated in graded ethanol, cleared in xylene, and mounted with neutral resin. Renal morphology was observed under a light microscope.

#### 2.10.2 Periodic acid-Schiff (PAS) staining

After fixation in 4% paraformaldehyde overnight, kidney tissues were dehydrated with a graded ethanol series, cleared in xylene, embedded in paraffin, and sectioned. Sections were dewaxed, rehydrated, and oxidized in 1% periodic acid for 15 min. After rinsing with distilled water, the sections were incubated with Schiff reagent for 20–30 min at room temperature in the dark. Following thorough washing in warm tap water, sections were counterstained with hematoxylin, dehydrated, cleared, and mounted with neutral resin. Renal basement membranes and glycogen deposition were evaluated under a light microscope.

### 2.11 Immunohistochemistry

Kidney tissues were washed with PBS and fixed with 4% paraformaldehyde. After antigen retrieval and PBS rinsing, the sections were incubated with the prepared primary antibody at 4°C overnight. Following extensive PBS washing, the secondary antibody was applied, followed by DAB staining. The tissues were then dehydrated through an ethanol gradient, cleared with xylene, and mounted with neutral resin. Finally, the stained sections were examined under a microscope.

### 2.12 Quantitative real-time polymerase chain reaction (RT-PCR) analysis

Total RNA was extracted using the Trizol method, and RNA concentration was measured before reverse transcription to cDNA. *β*-actin was used as the internal control. The amplification conditions were as follows: initial denaturation at 95°C for 30 s, followed by 40 cycles of 95°C for 15 s and 60°C for 30 s. The relative expression levels were calculated using the 2^^-ΔΔCt^ method. The premier used in this study included: human Klotho: forward 5′-GGGAGGTCAGGTGTCCATTG-3′, reverse 5′-TGCTCTCGGGATAGTCACCA-3′.human Lamin B1: forward 5′-CGCGTGCGTGTCTATGCTA-3′, reverse 5′-CCAACTGGGCAATCTGATCCT-3′.human PAI-1: forward 5′-GCAAGGCACCTCTGAGAACT-3′, reverse 5′-GGGTGAGAAAACCACGTTGC-3′.human p21: forward 5′-AGTCAGTTCCTTGTGGAGCC-3′, reverse 5′-CATTAGCGCATCACAGTCGC-3′.human p16: forward 5′-CCGAATAGTTACGGTCGGAGG-3′, reverse 5′-CACCAGCGTGTCCAGGAAG-3′.human *β*-actin: forward 5′-CACCCAGCACAATGAAGATCAAGAT-3′, reverse 5′-CCAGTTTTTAAATCCTGAGTCAAGC-3′.

### 2.13 Western blotting analysis

Total protein was extracted from cells and kidney tissues using RIPA (R401-01; Vazyme, Nanjing, China) lysis buffer. After complete lysis, the samples were centrifuged at 12,000 rpm at 4°C for 15 min. Protein concentrations were determined using a BCA assay, and the samples were denatured at 98°C for 13 min in a metal bath. Proteins were separated using SDS-PAGE with stacking and separating gels. Electrophoresis was conducted at 90 V for the stacking gel and 120 V for the separating gel, followed by transfer onto a PVDF membrane. The membrane was blocked with 5% nonfat milk at room temperature for 1 h and incubated with primary antibodies overnight at 4°C with gentle shaking. After recovering the primary antibodies, the membrane was washed three times with TBST. Secondary antibody incubation was performed at room temperature for 1 h, followed by TBST washes. Protein bands were visualized using ECL, and imaging was performed in a darkroom. The resulting images were analyzed using ImageJ software.

### 2.14 Statistical analysis

The results are expressed as the mean ± SEM. Statistical analysis was performed using SPSS 21.0 and GraphPad Prism 6.0 software. Experimental data conforming to a normal distribution were expressed as mean ± standard deviation (x̄ ± s). One-way analysis of variance (ANOVA) was used for comparisons among groups. If homogeneity of variance was met, post-hoc tests were conducted using the least significant difference (LSD) method; if not, the Tamhane’s T2 method was applied. *p* < 0.05 was considered significant.

## 3 Results

### 3.1 The protective effects of SYG on kidney injury in *db/db* mice

**Figure 1:**
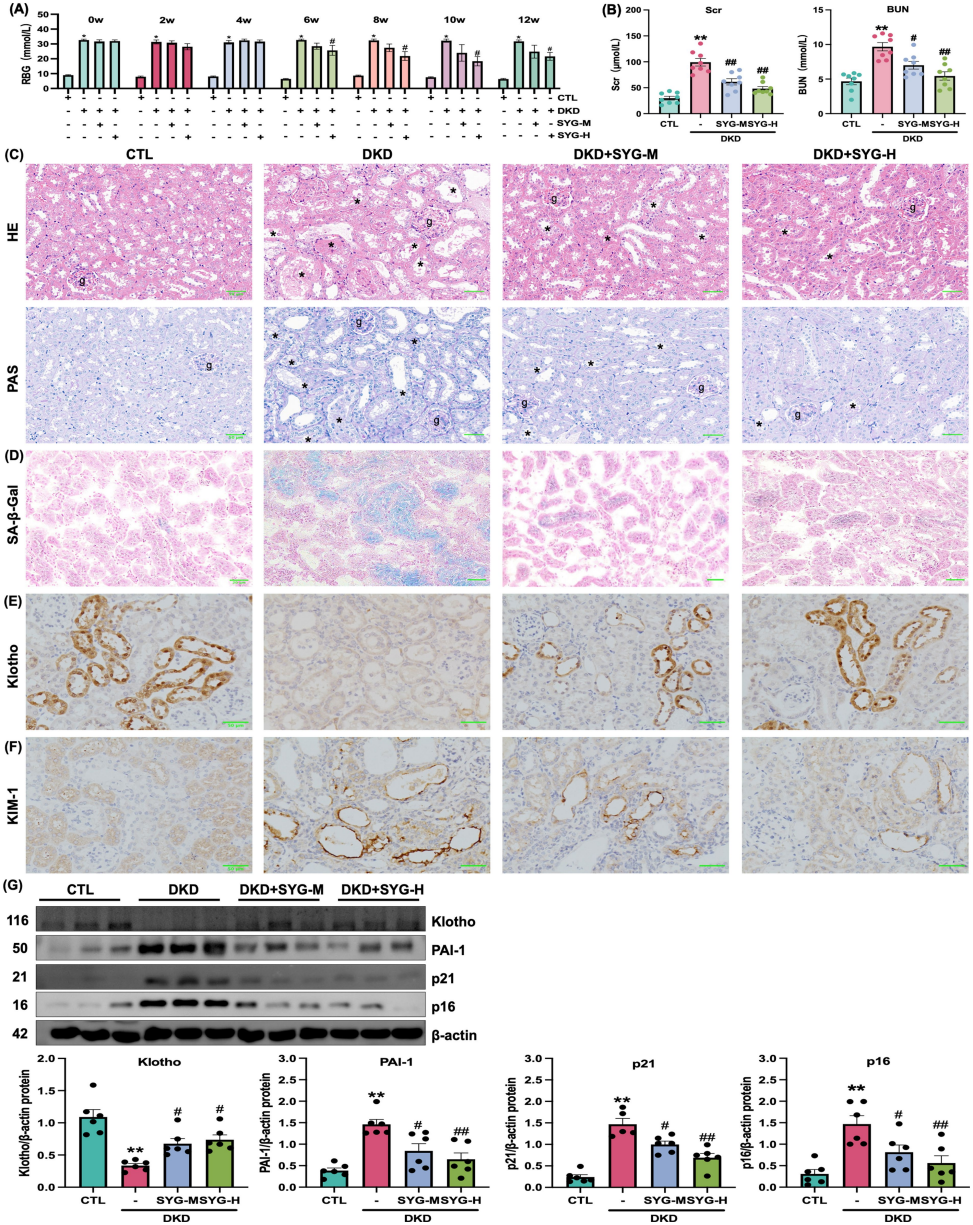
Renal protective effects of Shenyuan granules in adenosine-induced *db/db* diabetic kidney disease mouse model. **(A)** Effects of Shenyuan granules on blood glucose levels in different mouse groups (*n* = 8). **(B)** Levels of Scr and BUN across groups. **(C)** HE and PAS of kidney tissues from control, DKD, DKD + SYG-M, and DKD + SYG-H groups. **(D)** SA-β-Gal staining images showing senescent cells in kidney tissues across the groups. **(E,F)** Immunohistochemical detection of Klotho and KIM-1 expression in the kidneys of each group. **(G)** Quantitative analysis of protein expression levels of Klotho, PAI-1, p21, and p16 in kidney tissues in various groups. ^*^P < 0.05, ^**^P < 0.01 compared to the control group, *^#^P* < 0.05, *^##^P* < 0.01 compared to the DKD group (*n* = 6).

This study investigated the effects of different doses of SYG on kidney injury in *db/db* mice. The results showed that, compared with the DKD group, both doses of SYG significantly reduced random blood glucose levels in mice after 8 weeks of intervention (Figure 1A). Furthermore, both doses improved Scr and BUN levels, with the high-dose group showing more pronounced effects (Figure 1B). HE and PAS staining revealed clear and intact glomerular and tubular structures in the control group, with no pathological changes observed. In contrast, the DKD group exhibited tubular vacuolar degeneration, increased glomerular mesangial matrix, thickened basement membranes, tubular atrophy, interstitial fibrosis, and inflammatory cell infiltration. SYG treatment ameliorated these pathological changes, particularly in the high-dose group (Figure 1C). These findings indicate that SYG effectively alleviate kidney injury in *db/db* mice.

### 3.2 SYG inhibit cellular senescence in the kidney tissues of *db/db* mice

SA-*β*-Gal staining revealed that SYG significantly reduced the proportion of senescence-positive cells in kidney tissues compared with the model group (Figure 1D). Immunohistochemical staining further confirmed that Klotho was predominantly expressed in distal tubular epithelial cells and was significantly decreased in the DKD group. SYG treatment restored Klotho expression levels, particularly in the high-dose group (Figure 1E). Similarly, the expression of KIM-1, a marker of proximal tubular injury, was markedly elevated in the DKD group and was notably reduced following SYG intervention, indicating improved tubular integrity (Figure 1F). Additionally, SYG modulated the expression of senescence-related proteins by significantly downregulating the expression of p16, p21, and PAI-1 while upregulating the expression of Klotho (*p* < 0.05) (Figure 1G). In summary, SYG alleviated DKD progression by improving renal pathological damage and regulating cellular senescence-related signaling pathways.

### 3.3 Klotho overexpression alleviates AGE-induced senescence in HK-2 cells

**Figure 2:**
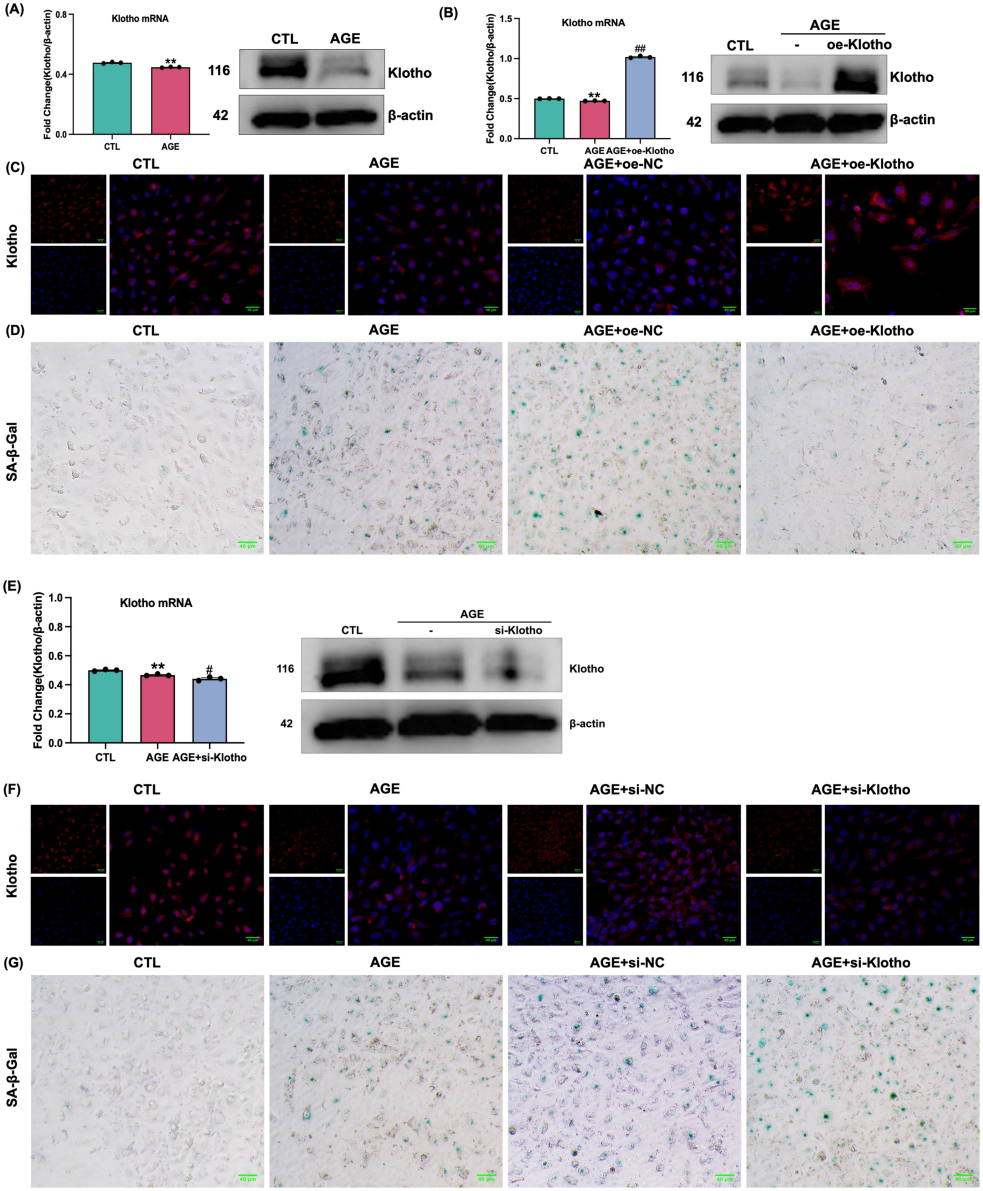
Validation of Klotho gene overexpression and silencing in AGE-induced HK-2 cells. **(A)** Changes in Klotho mRNA and protein expression in HK-2 cells following AGE stimulation. **(B)** Changes in mRNA and protein expression after Klotho overexpression in HK-2 cells. ^**^P < 0.01 compared to the control group, *^##^P* < 0.01 compared to the AGE group. **(C)** Klotho immunofluorescence images across the groups. **(D)** SA-β-Gal staining of the control, AGE, AGE + oe-NC, and AGE + oe-Klotho groups. **(E)** Changes in mRNA and protein expression in HK-2 cells following Klotho silencing. **(F)** Klotho immunofluorescence images across the groups. **(G)** SA-β-Gal staining and Klotho immunofluorescence images of various groups.

**Figure 3:**
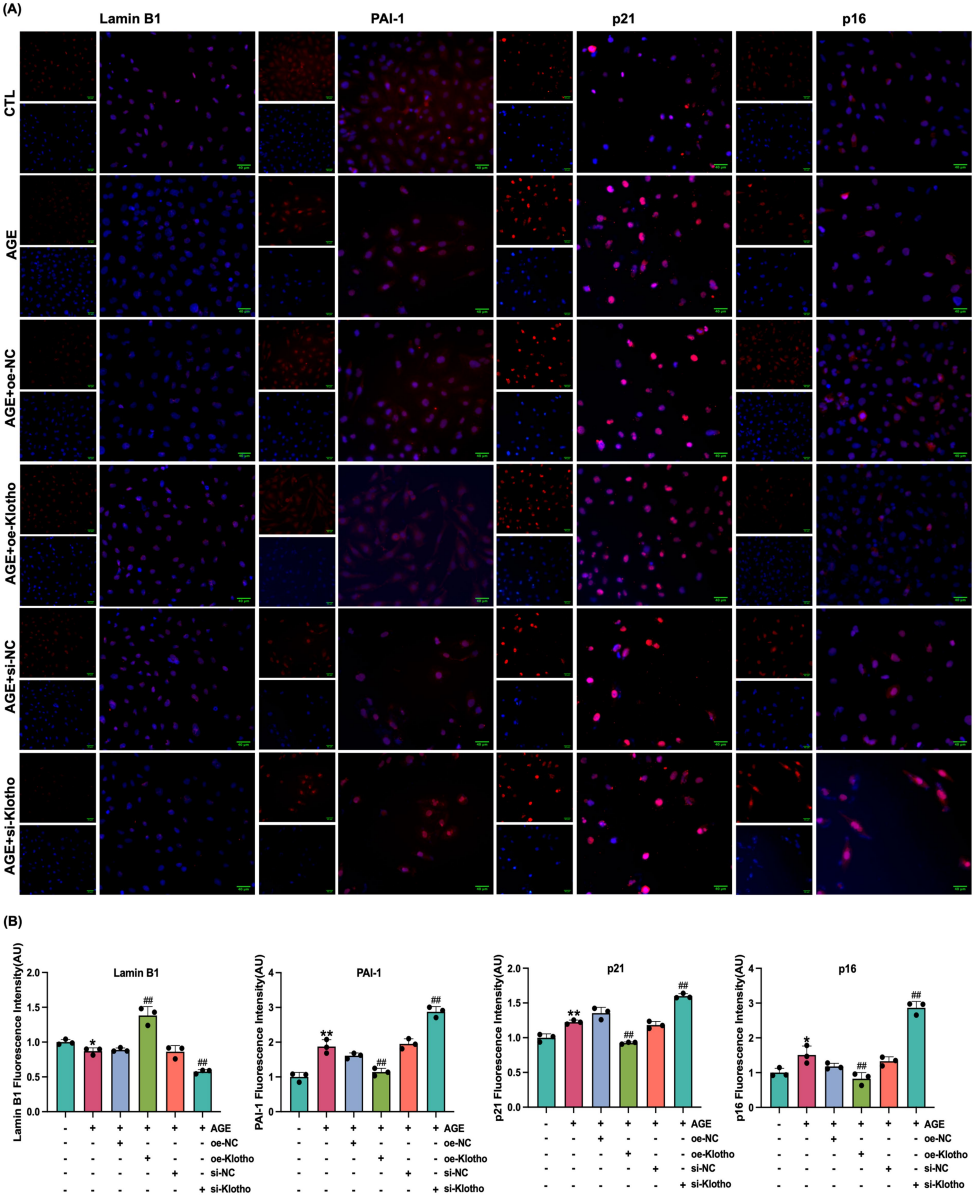
Klotho overexpression enhances Lamin B1 expression and inhibits PAI-1, p21 and p16 expression in AGE-induced HK-2 cells. **(A)** Immunofluorescence detection of Lamin B1, PAI-1, p21 and p16 expression in control, AGE, AGE + oe-NC, AGE + oe-Klotho, AGE + si-NC, and AGE + si-Klotho groups. **(B)** Quantitative immunofluorescence analysis of Lamin B1, PAI-1, p21 and p16 expression. ^*^P < 0.05, ^**^P < 0.01 compared to the control group, *^##^P* < 0.01 compared to the AGE group.

**Figure 4:**
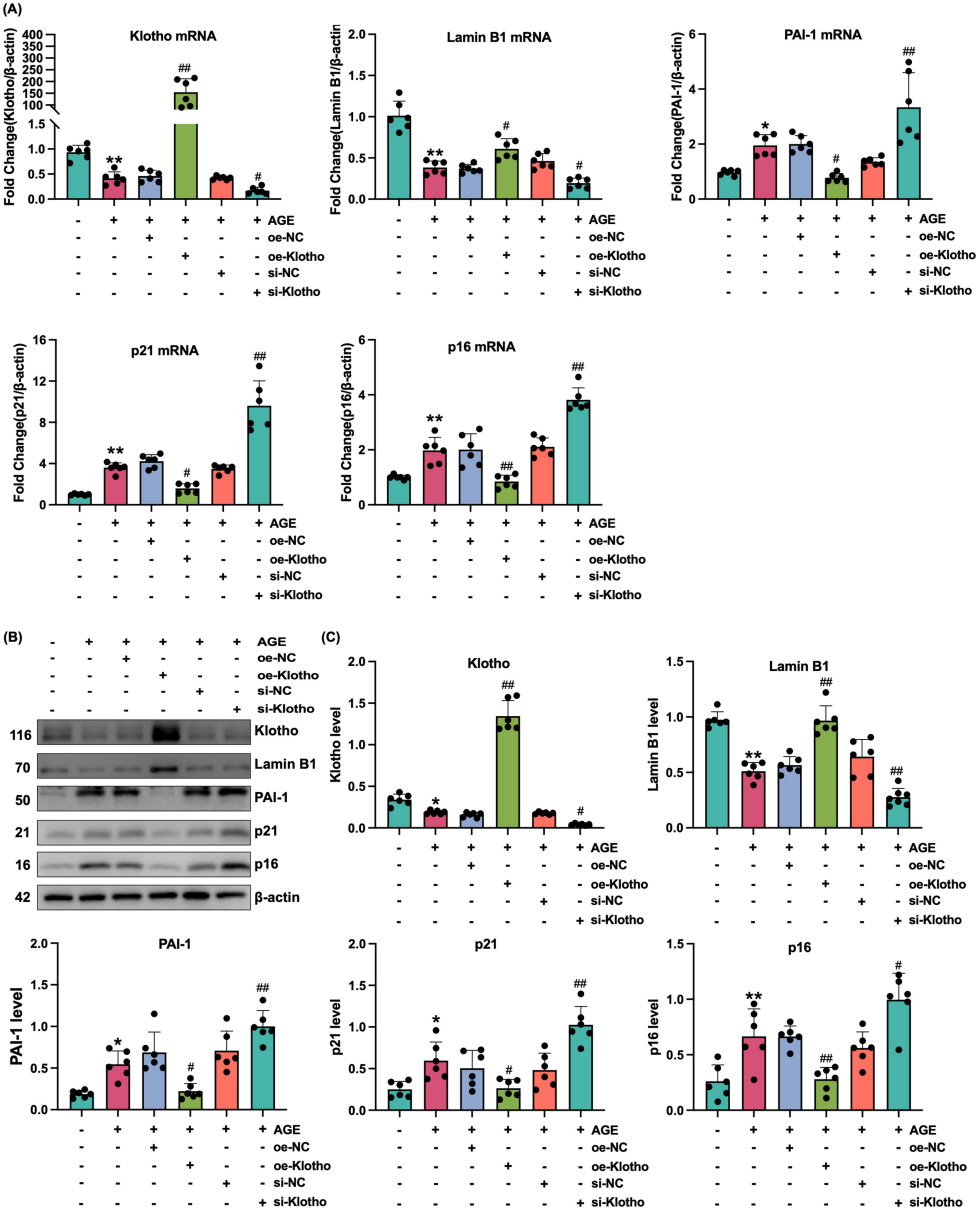
Klotho overexpression mitigates AGE-induced senescence in HK-2 cells. **(A)** mRNA expression levels of Klotho, Lamin B1, PAI-1, p21, and p16 in control, AGE, AGE + oe-NC, AGE + oe-Klotho, AGE + si-NC, and AGE + si-Klotho groups. **(B)** Protein expression of the same markers in the corresponding groups. **(C)** Detailed analysis of protein levels. ^*^P < 0.05, ^**^P < 0.01 compared to the control group, *^#^P* < 0.05, *^##^P* < 0.01 compared to the AGE group.

Due to reduced Klotho expression in the kidneys of *db/db* mice, this study explored the effects of Klotho overexpression and silencing on AGE-induced senescence in HK-2 cells. The results demonstrated that Klotho overexpression significantly increased Klotho gene and protein levels in HK-2 cells (*p* < 0.05) (Figures 2A–C), while reducing the proportion of SA-*β*-Gal-positive senescent cells (Figure 2D). si-Klotho-mediated knockdown of Klotho expression led to a significant increase in senescence markers, further supporting the critical role of Klotho in the senescence process (Figures 2E–G) Additionally, Klotho overexpression downregulated p16, p21, and PAI-1 mRNA and protein levels while upregulating Klotho and Lamin B1 expression (*p* < 0.05) (Figures 3, 4). These findings indicate that Klotho mitigates AGE-induced HK-2 cell senescence by regulating senescence-associated signaling molecules.

### 3.4 SYG inhibit AGE-induced senescence in HK-2 cells by regulating klotho

**Figure 5:**
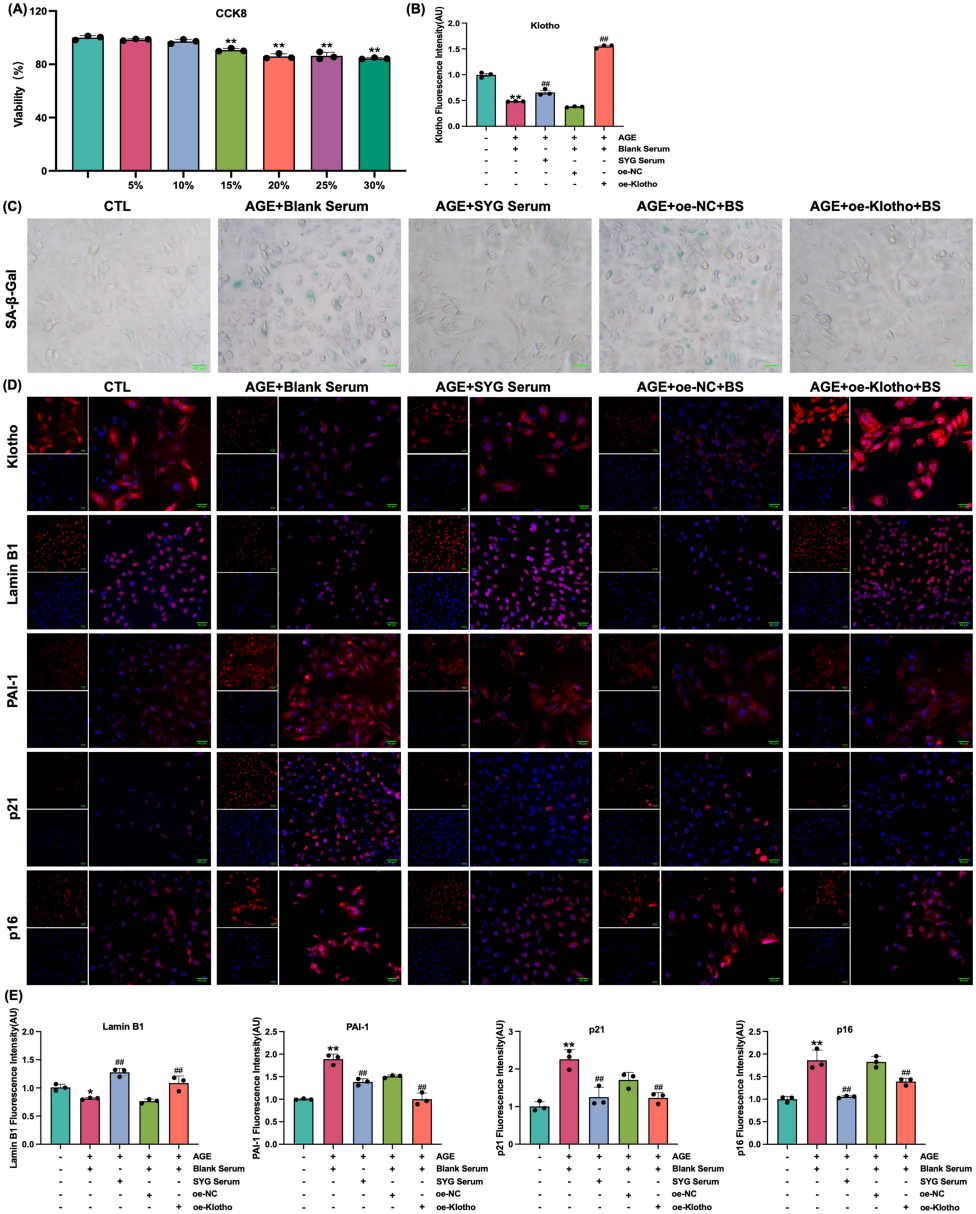
SYG enhance Klotho and Lamin B1 expression while inhibiting PAI-1, p21, and p16 expression in AGE-stimulated HK-2 cells. **(A)** Cell viability following treatment with varying concentrations of Shenyuan granules medicated serum (5–30%). **(B)** Quantitative immunofluorescence analysis of Klotho expression in control, AGE + Blank Serum, AGE + SYG Serum, AGE + Blank Serum + oe-NC, and AGE + Blank Serum + oe-Klotho groups. **(C)** SA-β-Gal staining of the same groups. **(D)** Immunofluorescence detection across the groups. ^**^P < 0.01 compared to the control group, *^##^P* < 0.01 compared to the AGE + Blank Serum group. **(E)** Quantitative immunofluorescence analysis of Lamin B1, PAI-1, p21 and p16 expression.

**Figure 6:**
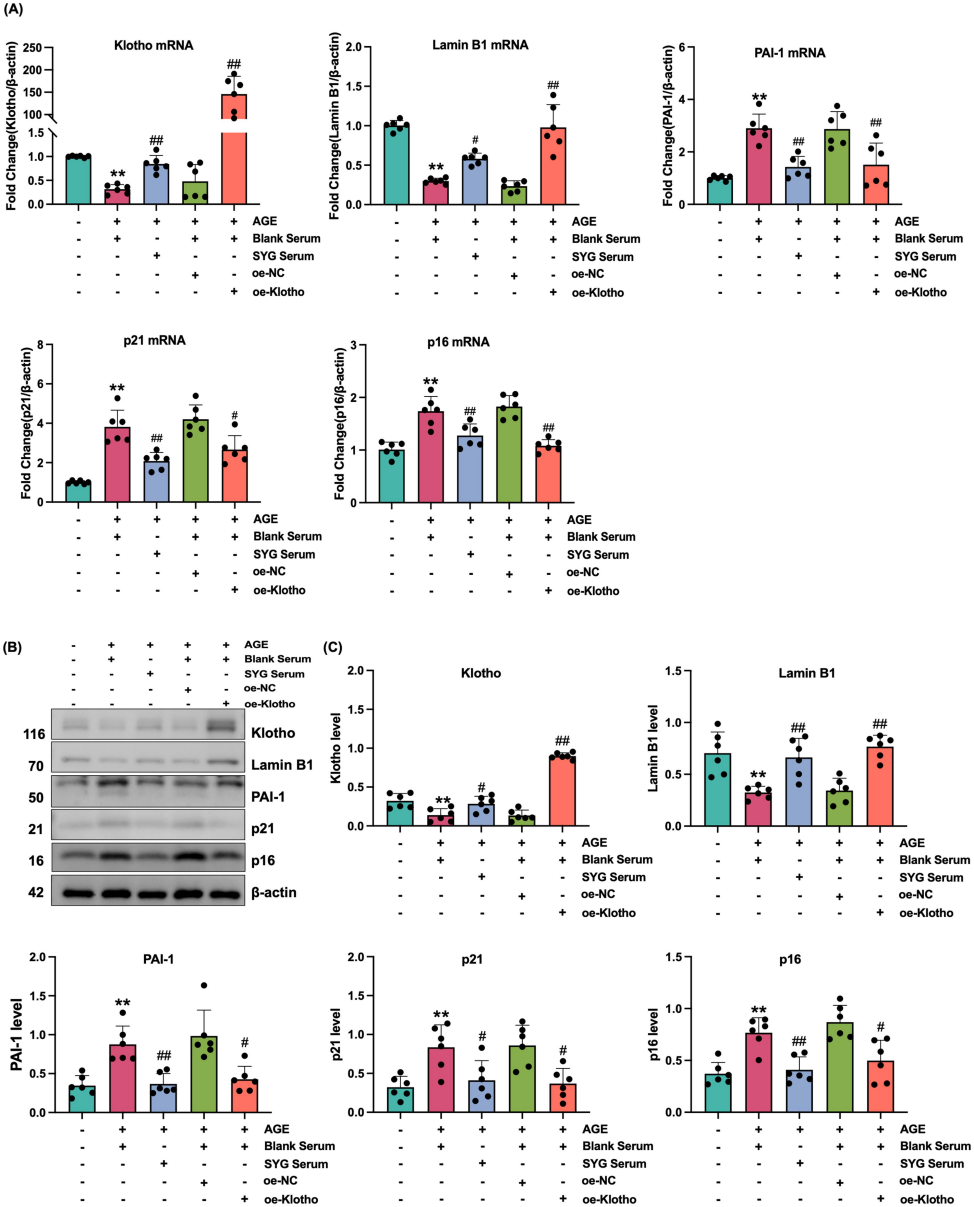
SYG mitigates AGE-induced senescence in HK-2 cells by enhancing Klotho expression. **(A)** mRNA expression levels of Klotho, Lamin B1, PAI-1, p21, and p16 in control, AGE + Blank Serum, AGE + SYG Serum, AGE + Blank Serum + oe-NC, and AGE + Blank Serum + oe-Klotho groups. **(B)** Protein expression of Klotho, Lamin B1, PAI-1, p21, and p16 in the same groups. **(C)** Detailed analysis of protein levels. ^*^P < 0.05, ^**^P < 0.01 compared to the control group, *^#^P* < 0.05, *^##^P* < 0.01 compared to the AGE + Blank Serum group.

This study explored the effects of drug-containing serum from SYG on AGE-induced senescence in HK-2 cells. CCK-8 analysis was used to determine the optimal concentration of drug-containing serum, and 10% SYG serum was selected for subsequent experiments due to its appropriate cytocompatibility (Figure 5A). SA-*β*-Gal staining showed a significant increase in the proportion of senescence-positive cells in the model group compared to the control group, which was significantly reduced after treatment with drug-containing serum (Figure 5C). Fluorescence analysis indicated increased expression of p16, p21, and PAI-1 and decreased expression of Klotho and Lamin B1 in the model group (*p* < 0.05). Treatment with SYG serum reversed these changes by decreasing p16, p21, and PAI-1 expression while increasing Klotho and Lamin B1 expression (*p* < 0.05) (Figures 5B,D,E). Consistent results were observed at the mRNA and protein levels: the model group exhibited elevated p16, p21, and PAI-1 mRNA and protein levels, along with reduced Klotho and Lamin B1 levels (*p* < 0.05). These changes were effectively reversed by SYG serum treatment (Figure 6). These findings demonstrate that SYG inhibit AGE-induced senescence in HK-2 cells by regulating Klotho and related senescence-associated molecules.

## 4 Discussion

Renal fibrosis, characterized by glomerulosclerosis and tubular interstitial fibrosis, is a hallmark pathological feature of chronic kidney disease (28–30). Previous studies on the mechanism of DKD have primarily focused on glomerular damage, with microalbuminuria (MAU) long regarded as an early marker of glomerular injury (31). However, recent epidemiological data reveal that 20.5–63.0% of diabetic patients experience renal dysfunction without MAU (32). Renal biopsy studies further suggest that tubular lesions in DKD are more prevalent and severe than glomerular damage (33–35). These findings underscore the need to reorient research efforts from glomerular to tubular mechanisms in diabetes-related kidney disease (36, 37).

Cellular senescence has emerged as a critical pathological mechanism in DKD (38–40). It refers to a state of irreversible cell cycle arrest triggered by stressors such as DNA damage or oxidative stress (41, 42). Recent studies have highlighted the therapeutic potential of TCM components showed anti-aging effects (43–46). In hyperglycemic conditions, AGEs act as metabolic toxins, triggering oxidative stress and chronic inflammation that exacerbate renal damage (47).

The Klotho gene, a key anti-aging regulator, exerts protective effects against DKD through antifibrotic, antiinflammatory, and antioxidant pathways (48). Some studies reported that TCM improved Klotho expression in a variety of diseases (49–51). The molecular pathways regulated by p16INK4a/Rb and p53/p21 are pivotal in cellular senescence (52). p16, encoded by the CDKN2A gene, inhibits cell cycle progression and is closely associated with senescence (53). p21 negatively regulates the cell cycle by inhibiting CDK activity (54). In hyperglycemia, demethylation of the p21 promoter accelerates its expression, triggering the SASP, characterized by increased inflammatory and chemokine factors, thereby impairing tubular repair and promoting interstitial fibrosis (55). PAI-1, a reliable marker of senescence, is associated with renal fibrosis, fibrin deposition, and cellular senescence, particularly in Klotho-deficient mice (56). Lamin B1, a nuclear lamina protein essential for nuclear integrity, is closely related to cellular senescence and DNA damage responses (57). This study demonstrated that Klotho overexpression significantly increased Lamin B1 levels, reduced p16, p21, and PAI-1 expression, and decreased the proportion of SA-*β*-Gal-positive cells in HK-2 cells exposed to AGE, underscoring Klotho’s protective role against senescence in hyperglycemia.

Accumulating evidence supports the application of TCM in treating various renal disorders, including DKD (58–62). Recent studies have highlighted the therapeutic potential of TCM components in improving DKD through anti-aging mechanisms (63). Astragaloside IV, a primary active component of *Astragalus membranaceus*, has been shown to upregulate Klotho expression by inhibiting the NF-κB/NLRP3 axis, thereby alleviating high-glucose-induced podocyte injury and offering renoprotective effects in DKD (64, 65). Similarly, the combination of Astragalus and Rheum was demonstrated to improve DKD pathology through multi-target, multi-pathway interactions, providing evidence of their synergistic efficacy (66). Furthermore, Epimedium and its bioactive component, icariin, are known for their kidney-tonifying properties, which align with their potential to regulate cellular senescence and oxidative stress (67). These findings support the hypothesis that SYG, composed of Astragalus, Rheum, and Epimedium, may exert their anti-senescence and renoprotective effects in DKD by modulating the Klotho-mediated p16/p21 signaling pathway, providing a mechanistic basis for their clinical use.

In recent years, senescence-targeted therapies have garnered increasing attention as potential interventions for chronic diseases and age-related pathologies. These therapeutic strategies are broadly classified into two categories: senolytics and senomorphics (68, 69).

Senolytics are agents that selectively induce apoptosis in senescent cells, thereby reducing their pathological burden. Representative compounds include dasatinib, a tyrosine kinase inhibitor, and quercetin, a plant-derived flavonoid. The combination of these two agents (D + Q) has been shown to effectively eliminate senescent endothelial cells, preadipocytes, and osteoblasts *in vitro* and *in vivo* (70). Notably, to date, D + Q remains the only senolytic combination that has demonstrated functional benefits in early-phase human clinical trials, including improved physical function in diabetic kidney disease (71). Other potent senolytics include navitoclax (ABT-263), which targets BCL-2/BCL-xL anti-apoptotic signaling, but it has shown dose-limiting thrombocytopenia in preclinical and early clinical trials (72–75).

Senomorphics, in contrast, do not eliminate senescent cells but instead suppress the SASP and modulate downstream signaling pathways. Agents like rapamycin, metformin, and JAK inhibitors have shown efficacy in reducing SASP-related inflammation and improving tissue function without inducing cell death (76, 77). However, most senomorphics exert broad signaling effects and may not selectively modulate aging-related pathways, raising concerns about off-target immunosuppression, metabolic disturbance, or long-term toxicity.

While these emerging agents hold promise, they remain largely in preclinical or early clinical stages, with limited long-term safety data and no approved senescence-targeting indication thus far. Importantly, senolytics often derive from anti-cancer drugs and exhibit significant toxicity, including hematologic suppression, hepatic impairment, and the risk of disrupting physiological senescence involved in wound healing and tissue remodeling (78, 79).

By contrast, SYG represent an already-approved clinical formulation with established safety and efficacy in traditional medicine practice. Our previous studies, together with current experimental findings, indicate that SYG may exert anti-senescent effects through endogenous pathways, particularly via the restoration of Klotho expression, a key anti-aging protein known to regulate oxidative stress, Wnt signaling, and cellular senescence (13, 20, 80, 81). Unlike senolytics that act through cytotoxicity or senomorphics that rely on broad pathway suppression, SYG may function through a gentler, homeostatic mechanism, enhancing intrinsic cellular resilience against stress-induced senescence. This unique mode of action suggests that SYG may offer a safer and more sustainable anti-senescence strategy, particularly for long-term management of chronic conditions such as DKD.

Nevertheless, we recognize that further comparative and mechanistic studies are needed to rigorously define the distinctions between SYG and conventional senotherapeutics, both in efficacy and safety profiles, and to elucidate the translational potential of SYG as a complementary or alternative approach to modulating cellular senescence.

## 5 Conclusion

In summary, SYG effectively improve renal function and delay cellular senescence, potentially through the Klotho-mediated p16/p21 pathway. However, this study primarily analyzed protein, transcriptional, and pathological levels. Future research will explore genetic and metabolic dimensions to provide a more comprehensive theoretical basis for the prevention and treatment of DKD using TCM. Targeting senescent cells may offer a promising strategy for treating renal diseases and preventing DKD.

## Data Availability

The original contributions presented in the study are included in the article, further inquiries can be directed to the corresponding author.
